# The Effects of Website Traits and Medical Skepticism on Patients’ Willingness to Follow Web-Based Medical Advice: Web-Based Experiment

**DOI:** 10.2196/29275

**Published:** 2022-02-18

**Authors:** Jennifer Claggett, Brent Kitchens, Maria Paino, Kaitlyn Beisecker Levin

**Affiliations:** 1 School of Business Wake Forest University Winston-Salem, NC United States; 2 McIntire School of Commerce University of Virginia Charlottesville, VA United States; 3 Department of Sociology, Anthropology, Social Work, and Criminal Justice Oakland University Rochester, MI United States; 4 WholeHealth Medical Charlottesville, VA United States

**Keywords:** web-based information credibility assessment, website traits, medical skepticism, mobile phone

## Abstract

**Background:**

As people increasingly turn to web-based sources for medical information, we offer some insight into what website traits influence patients’ credibility assessment. Specifically, we control for brand and content length, while manipulating three website traits: authorship, format, and tone. Furthermore, we focus on medical skepticism to understand how patients with high levels of medical skepticism may react to web-based medical information differently. Medical skepticism is related to a patient’s doubts about the value of conventional medical care; therefore, skeptics may have different practices and criteria when conducting their own web-based medical searches.

**Objective:**

The aim of this study is to evaluate how website traits affect the likelihood that patients follow web-based medical advice and how this varies among patients with differing levels of medical skepticism.

**Methods:**

This web-based experiment presented participants with a hypothetical medical situation about leg cramps and offered a website with treatment advice. We varied the websites the participants observed across three traits: authorship (patient or physician), format (article or discussion forum), and tone (objective or experience-based). The 2305 participants were randomly assigned to 1 of 8 possible conditions and then asked the extent to which they would follow the advice. Health care patterns and coverage, demographics, and the participants’ level of medical skepticism were captured.

**Results:**

Our participants were selected to be demographically representative of the population of internet users in the United States. The 2305 complete responses were analyzed with ordinary least squares regression. Our analysis reveals that people are more likely to accept web-based medical advice authored by a physician (*P*<.001) and presented with an objective tone (*P*=.006), but these preferences erode as the levels of medical skepticism increase. Medical skepticism was measured by means of a previously established index on a 0 to 4 scale, and the average score was 2.26 (SD 0.84). Individuals with higher levels of medical skepticism were more likely to follow web-based medical advice in our experiment (*P*<.001). Individuals with low levels of medical skepticism found the discussion forum format more credible, whereas those with high levels of medical skepticism preferred the article format (*P*=.03). We discuss the interactions between medical skepticism and all 3 website traits manipulated in the experiment.

**Conclusions:**

Our findings suggest that, generally, physician authorship and an objective tone create more persuasive web-based medical advice. However, there are differences in how patients with high levels of medical skepticism react to web-based medical resources. Medical skeptics are less discerning regarding the author’s credentials and the presentation tone of the information. Furthermore, patients with higher levels of medical skepticism prefer article format presentations, whereas those with lower levels of medical skepticism prefer discussion forum–style formatting.

## Introduction

### Background

Skepticism toward medical care and treatment has been a long-standing issue in health care [1], ranging from the development of antivaccination beliefs [2,3] and affecting how smokers perceive health-risk information [4] to affecting which treatments people with arthritis decide to use [5]. Medical skepticism, specifically, is related to a patient’s doubts about the value of medical care and is defined as “global doubts regarding the ability of conventional medical care to appreciably alter health status” and has been shown to correlate with higher rates in mortality at follow-up [6]. However, other studies have noted the potential benefits of medical skepticism, such as medically skeptical older adults reporting higher levels of self-rated overall health [7]. Previous work has focused on which demographics are more inclined to be skeptical of health care, generally concluding that it is highest among young White people with less education and often lacking health insurance [8,9]. Even so, medical skepticism is widespread, and the trend may be rooted in a consumerist movement where patients are encouraged to be more involved in their health care, making it of particular interest to understand how their decision-making process regarding medical care unfolds [10].

The internet is an obvious tool for patients to find alternative medical information [11,12]. All patients, regardless of their medical skepticism, increasingly use web-based resources to seek health information [13,14]. A Pew Research Center report found that 80% of US internet users (>93 million people) have searched for health-related topics on the web, and most of the health-related searches are related to specific medical problems they are experiencing [15]. In 1 survey, 80% of the physicians confirmed experiences of patients presenting internet-sourced medical information [16]; and in another survey, 41% of the patients stated that they had used a medical professional to confirm their own internet diagnoses [15]. However, this growing interest in web-based medical information has been answered by an explosion of available health content, ranging from predatory websites that spread misinformation and nonprofit institutions creating web-based article repositories to patients using interaction-enabled web-based formats to discuss medical information in a question-and-answer format [17-19]. As gathering health information on the web has been established as a norm, it is important that we understand what causes a patient to follow or ignore medical advice they read on the web [20-24]. Logically, medically skeptical patients may also have different web search behaviors and react to website traits differently. Therefore, we consider what web-based content traits drive people’s willingness to follow the advice, as well as the role medical skepticism plays in the web-based credibility assessment process.

Previous research has looked at the use of patient portals as a web-based form of communication between patients and physicians [25-27], but portals provide a web-based communication mechanism that supplements a previously established relationship between a physician and a patient. Prior research has also considered how active participation in web-based health care communities may influence patient outcomes and provide emotional support [28-31]; yet, this assumes that a patient has joined, and is actively participating in, a community, usually sharing their own details and questions. In contrast, our study aims to better understand how patients decide whether to follow web-based medical advice in more casual web research scenarios, specifically those that do not necessarily require the intervention of a physician or long-term emotional support. To that end, we focus on three potential traits that shape web content and may affect credibility assessment: authorship, format, and tone of presentation.

The objectives of this study are to (1) determine what website traits affect a patient’s likelihood to follow the presented medical advice and (2) determine whether patients with higher levels of medical skepticism exhibit different patterns of website traits influencing their likelihood to follow medical advice found on the web. Our findings increase our understanding of how patients decide whether to follow web-based medical advice and may inform the design of health care websites. Furthermore, we extend our knowledge about medical skepticism by finding evidence that it alters the credibility assessment that patients undertake when considering web-based medical advice.

### Website Traits

#### Overview

There has been an explosion in medical websites in the last several decades that individuals may turn to for obtaining advice, sharing experiences, voicing concerns, and informing decisions [32-34]. In any context, web users must look for cues signaling that the content is credible and trustworthy [35,36]. We identify three specific website traits that vary across common health information websites: authorship, format, and presentation tone.

#### Authorship

The authorship of content is a major factor in how people assess the credibility of information [37]. A meta-analysis of studies looking at the effects of health care expertise on the credibility of health information suggests that authorship of web-based content is important and experts are favored [38]. However, the research is not consistent, and in some situations, patients prefer nonexpert advice [39] or perceive a source as credible because of positive judgments about the trustworthiness of the author instead of professional credentials [21]. For simplicity, we focused on two major categories of authors who often provide web-based health care information: physicians and other patients. Physicians represent the easy-to-recognize role of a health care professional who has formal training and expertise, whereas other patients are peers who may have faced similar medical situations.

#### Format

The increasing prevalence of user-generated content is one of the strongest trends of the past decade [40,41]. This has created new types of web-based content fundamentally different from the more traditional static webpage format, especially regarding navigation design and connectedness [42]. For example, a medical article is usually presented as a static page and is read-only. The owner of a static webpage is also the controller of the information presented. For brevity, we refer to this static, read-only delivery as article format. In contrast, web forums or blogs invite users to also create and share content (eg, ask questions or respond to previous posts) in a dynamic and participative manner [41]. This cocreation process means that the webpage owner is not solely responsible for generation of the content that is visible. We refer to this dynamically cocreated content as discussion forum format. The article format presents a single point of view in a controlled environment, whereas the discussion forum format often presents a cocreated set of advice.

#### Tone of Presentation

Medical information can be presented on the web as objective or experiential [9]. Objective information is content presented as fact, devoid of any personal attachment or interpretation by the person conveying the information. Experiential information is presented as derived from the actual experiences and insights of the person conveying the information. Some previous research makes the assumption that information provided by health care professionals is objective and information provided by other patients is experiential [28]; however, we believe that the 2 should not be conflated but instead considered separately. Health care professionals can offer advice because of their experiences with other patients, and other patients can convey information as objective fact. Broadly speaking, people tend to appreciate experiential information in decision-making contexts because it signals familiarity with the content [21]; yet, traditionally, an objective presentation signals that the information is well established and broadly accepted [43].

## Methods

### Sampling and Data Collection

We conducted a web-based experiment through a web-based survey. Qualtrics software was used to recruit participants, and after excluding those with missing or incomplete data, we obtained a sample of 2305 participants. Panelists were recruited to be demographically representative of the population of internet users in the United States, which is a strength of Qualtrics panels [44]. We excluded participants who worked in a medical profession and those who had previous experience with leg cramps (the focus of our experimental manipulation) to remove participants with previous expertise in the study’s context. We captured demographics (sex, race, education level, income, age, and geography: whether they lived in a rural, suburban, or urban area), health care situation (number of recent health care visits, current method of receiving primary care, and health insurance status), and the respondent’s level of medical skepticism.

Each participant was presented with the same hypothetical medical situation to begin the experiment: “Your friend has recently been battling leg cramps. They ask you to help them research the condition online, and you find the following resource. Please read the web page presented and then answer the questions related to your experience with the online resource.”

This situation was chosen such that there would be a nontrivial treatment recommendation that could nonetheless be administered without formally seeking professional medical intervention. Next, 1 of 8 different webpages containing health information regarding leg cramps was presented to the participant. The set of 8 webpages used in the experiment (2×2×2 experimental design) represents each possible permutation of the three website traits of interest: authorship (physician or patient), format (article or discussion forum), and tone (objective or experiential). Each prompt provides identical treatment advice regarding leg cramps, regardless of the website traits shown in the experimental manipulation to which they were assigned.

**Figure 1:**
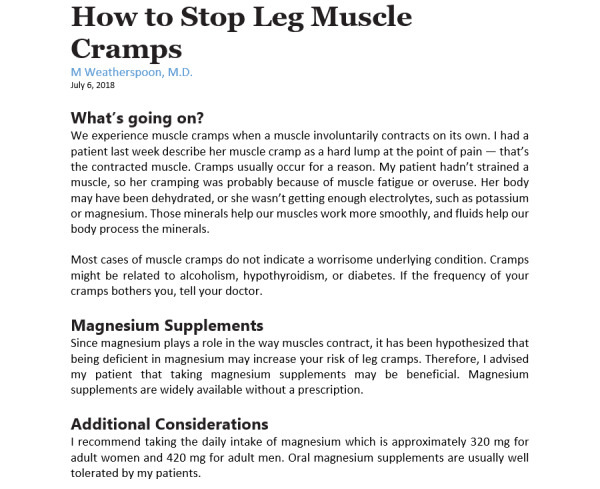
Experimental website prompt displaying physician authorship, article format, and experiential presentation tone.

**Figure 2:**
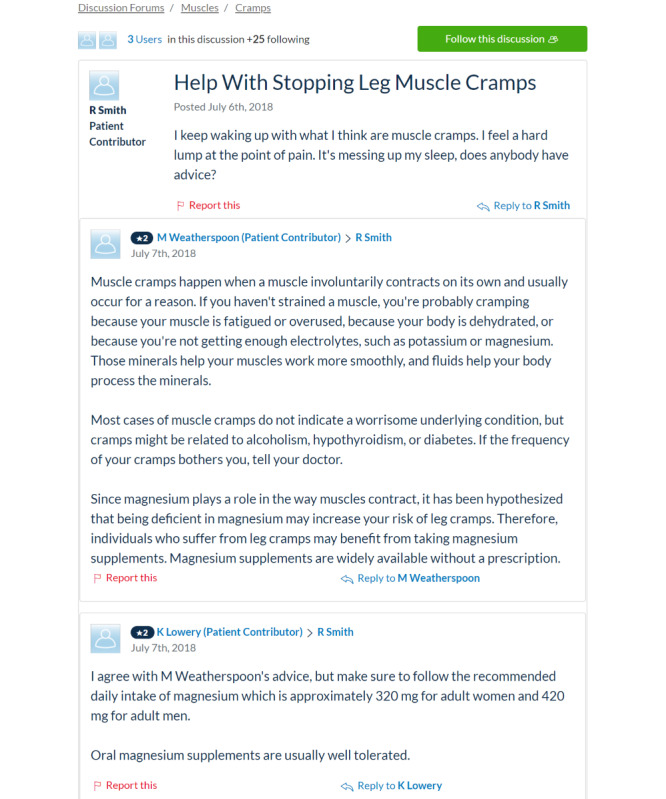
Experimental website prompt displaying patient authorship, discussion forum format, and objective presentation tone.

A positive perception of a specific brand or the reputation of a website increases the likelihood that people will follow the presented advice [42,45-47]. Opinions of friends and acquaintances about a particular website source might also influence patients because they build brand recognition [48,49]. In addition, the length of the text and other formatting features that affect the ease of skimming (eg, bullet points vs long blocks of text) are known to affect the likelihood that people will read the advice and then follow that advice [50-52]. To avoid confounding effects rooted in brand recognition, text length, or readability, our experimental design eliminates these factors by removing branding and standardizing the text length and grammatical presentation of the content shown to participants across all 8 website prompts (including order of information and sentence structure to the extent possible). Example prompts are presented in Figures 1 and 2; all 8 are available upon request.

Individuals were randomly assigned to each experimental condition, and there were between 269 and 301 individuals in each of the 8 possible website version groups. After viewing the prompt, participants were asked to indicate the likelihood that they would recommend the presented advice to their friend with leg cramps.

### Outcomes and Measures

#### Follow Web-Based Medical Advice

Our dependent variable was a continuous variable, ranging from 0 to 4, where lower numbers indicated that individuals were less likely to follow web-based medical advice and higher numbers indicated that individuals were more likely to follow web-based medical advice. This variable was an index composed of four ordinal measures indicating the extent to which individuals agreed with the following: (1) recommending that their friends try magnesium supplements, (2) the extent to which individuals believe that trying magnesium supplements is good advice, (3) if an individual had leg cramps, the extent to which they agree that they would try magnesium supplements, and (4) the webpage convinced them that they should try magnesium supplements to reduce leg cramps.

Individuals reported the extent to which they agreed with each of these statements by responding to a Likert scale that ranged from *strongly disagree* to *strongly agree*. We averaged these measures to form a continuous index for following web-based medical advice (Cronbach α=.94).

#### Independent Variables

##### Experimental Conditions

We included three independent variables to account for each of the aforementioned experimental conditions: the author (physician or patient), the format (article or discussion forum), and the tone (objective or experiential).

##### Medical Skepticism

We used an index to measure medical skepticism, and this index was based on 3 characteristics that captured an individual’s skepticism toward medicine. Medical skepticism was measured by means of a previously validated scale [8,53]. Respondents were asked the extent to which they agreed (on a scale of 0-4) with the following questions: (1) I can overcome most illness without help from a medical professional, (2) home remedies are better than medicines prescribed by doctors, and (3) I understand my health better than most physicians do (Cronbach α=.62). Although the reliability score is below the recommended threshold of Cronbach α=.70, it is an established scale [8,53] and similar to the reliability score reported in previous studies (eg, Cronbach α=.69 in the study by Fiscella et al [6] and Cronbach α=.63 in the study by Jensen et al [10]).

##### Health Care Situations

We used several independent variables to account for individuals’ health care situations. These were important aspects to capture for both our research objectives. A patient’s medical care situation and exposure may influence how they interpret web-based health care information [54,55], in general, and previous work has noted that medical skeptics may seek out in-person health care differently [8]. To accurately measure and control for these variations, we first included the approximate number of health care visits that respondents had made in the last 2 years because of illness or injury. Health care visits included visits that respondents have made for themselves or as a companion to a friend or family member because either visit would expose the individual to conversations with individual health care providers. Second, we used a measure that indicated the location where respondents sought primary medical care. This was a dichotomous measure that showed whether a respondent used emergency or urgent care as their primary care or whether they used a clinic, primary care physician, or other health care facility or whether the respondent did not seek any medical care. Finally, we included a variable that measured whether the respondent had health insurance. We compared those who did not have health insurance with individuals who had any type of health insurance (ie, Medicaid, Medicare, employer-provided, military or veteran, self-funded).

#### Control Variables

We included geographical setting and demographic data in our models, including sex, race, age, income, and educational attainment (Table 1).

**Table 1:** Descriptive statistics (N=2305).

Variable		Description	Value
Dependent variable: follow web-based medical advice, mean (SD)		This is a continuous variable, ranging from 0 to 4, indicating the likelihood that the respondent will follow the web-based medical advice provided at the end of the prompt (Cronbach α=.94)	2.97 (0.90)
**Experimental conditions: 2 × 2 × 2 design involving the following three factors, n (%)**			
	Physician	Dichotomous variable indicating that the experimental prompt was written by a physician (as opposed to a patient)	1170 (50.76)
	Article	Dichotomous variable indicating that the experimental prompt was written in article format (as opposed to a discussion forum format)	1171 (50.8)
	Objective	Dichotomous variable indicating that the experimental prompt was written in an objective manner (as opposed to experiential manner)	1158 (50.24)
Medical skepticism, mean (SD)		This is a continuous variable, ranging from 0 to 4, indicating the extent to which the respondent is skeptical toward medicine (Cronbach α=.62)	2.26 (0.84)
**Health care situation**			
	Number of health visits, mean (SD)	The approximate number of health care visits in the last 2 years (as a patient or accompanying a friend or family member)	4.40 (3.47)
	Primary care is emergency or urgent, n (%)	Dichotomous variable indicating that the respondent uses emergency or urgent care as their primary or usual care option (as opposed to other sources such as primary care physician or a community clinic)	477 (20.69)
	No insurance, n (%)	Dichotomous variable indicating that the respondent has health insurance (as opposed to being without insurance)	344 (14.92)
**Home geographical category, n (%)**			
	Rural	Respondent lives in a rural setting	558 (24.21)
	Suburban	Respondent lives in a suburban setting	1046 (45.38)
	Urban	Respondent lives in an urban setting. This is the reference category	701 (30.41)
**Demographics**			
	Female, n (%)	Respondent is female (the reference category is male)	1352 (58.66)
	Asian or Pacific Islander, n (%)	Respondent identifies as Asian or Pacific Islander	158 (6.85)
	Black, n (%)	Respondent identifies as Black	511 (22.17)
	Hispanic or Latino, n (%)	Respondent identifies as Hispanic or Latino	210 (9.11)
	Other race, n (%)	Respondent identifies as other race	88 (3.82)
	White, n (%)	Respondent identifies as White. This is the reference category	1338 (58.05)
	Bachelor’s degree, n (%)	Dichotomous variable indicating that the respondent has at least a bachelor’s degree (reference category is less than a bachelor’s degree)	689 (29.89)
	Income, mean (SD)	An ordinal variable, ranging from 0 to 11, entered into the model as a continuous predictor because of its underlying interval–ratio nature. The answer 4 (the average) indicates a salary of approximately US $30,000 to US $39,999	4.01 (3.16)
	Young, n (%)	Respondent is young: aged 18-34 years	728 (31.58)
	Middle-aged, n (%)	Respondent is middle-aged: aged 35-64 years	1223 (53.06)
	Older, n (%)	Respondent is older: aged 65 to ≥85. This is the reference category	354 (15.36)

### Data Analysis

We used an ordinary least squares (OLS) regression model to analyze how factors such as the experimental condition, medical skepticism, and demographic characteristics contributed to the extent to which individuals were willing to follow the advice offered for a leg cramp medical condition. Our dependent variable was continuous in nature, thus making OLS regression the most appropriate analytical model.

### Ethics Approval

This study was approved by the University of Virginia's Institutional Review Board for Social and Behavioral Science (Project Review 2018-0398-00).

## Results

### Participant Characteristics

Of the 2305 participants in our study, 1352 (58.66%) were women. Our sample was racially diverse, with 58.05% (1338/2305) of the participants identifying as White, 22.17% (511/2305) as Black, 9.11% (210/2305) as Hispanic or Latino, 6.85% (158/2305) as Asian or Pacific Islander, and 3.82% (88/2305) as a different race. Of the 2305 participants, 1223 (53.06%) were aged 35-64 years and 728 (31.58%) were aged 18-34 years, whereas 354 (15.36%) were aged >65 years.

On average, the respondents reported approximately 4.40 (SD 3.47) health care visits in the last 2 years (to accompany a friend or family member or as patients themselves), and 20.69% (477/2305) of the respondents indicated that their primary care occurred in an emergency room or urgent care clinic. Most of our respondents carried some sort of insurance (eg, Medicare, Medicaid, and private insurance), but 14.92% (344/2305) reported having no insurance provider. Our respondents included those who lived in suburban (1046/2035, 45.38%), urban (701/2305, 30.41%), and rural (558/2305, 24.21%) areas. Of the 2305 respondents, 689 (29.89%) had a bachelor’s degree or higher, and the average respondent reported an income generally consistent with that of the middle class [56] (Table 1).

### Following Web-Based Medical Advice

At the end of each prompt, the respondents were provided with the same treatment advice regarding leg cramps, regardless of the website traits shown in the experimental manipulation. We present OLS regression results (Table 2) that assess how experimental conditions and medical skepticism contribute to the likelihood that respondents will follow this advice. In model 1 (Table 2), we focus on the direct relationships between our independent variables of interest and the likelihood that respondents follow web-based medical advice. Our findings suggest that website traits do affect whether individuals are likely to follow medical advice. When presented with an example written by a physician, the respondents were significantly more likely to follow advice (*P*<.001). Similarly, an objective writing style, as opposed to a tone reflecting personal experience, was positively associated with following web-based medical advice (*P*=.006).

**Table 2:** Ordinary least squares regression analysis of following web-based medical advice regressed on experimental conditions.

			Model 1	*P* value	Model 2	*P* value
**Experimental conditions**						
	Physician author		0.184	<.001	0.385	<.001
	Article format		0.019	.60	–0.196	.06
	Objective writing style		0.101	.006	0.312	.003
	Medical skepticism		0.099	<.001	0.144	.001
**Interactions**						
	Skepticism × physician		—^a^	—	–0.089	.04
	Skepticism × article		—	—	0.094	.03
	Skepticism × objective		—	—	–0.094	.03
**Health care**						
	Number of health care visits		0.025	<.001	0.025	<.001
	Primary care is emergency or urgent		0.099	.04	0.094	.047
	No insurance (reference: any insurance)		–0.018	.73	–0.012	.83
**Control variables**						
	**Setting (reference: urban setting)**					
		Rural	–0.072	.18	–0.074	.16
		Suburban	–0.009	.84	–0.009	.84
	Female		0.010	.80	0.013	.73
	**Race (reference: White)**					
		Asian	–0.114	.14	–0.121	.12
		Black	–0.042	.40	–0.045	.36
		Hispanic	0.015	.83	0.019	.78
		Other race	–0.075	.44	–0.079	.42
	Bachelor’s degree or higher (reference=less than bachelor’s degree)		–0.232	<.001	–0.225	<.001
	Income		0.007	.28	0.007	.31
	**Age (years; reference: older adults aged ≥65 years)**					
		Young (18-34)	–0.244	<.001	–0.242	<.001
		Middle-aged (35-64)	–0.121	.03	–0.120	.03
	Constant		2.678	<.001	2.576	<.001
*R*^2^ (N=2305)			0.059	N/A^b^	0.060	N/A

^a^Not included in base model.

^b^N/A: not applicable.

As medical skepticism increased, individuals were more likely to follow web-based medical advice (*P*<.001). This may be because these individuals are more receptive to self-service sources of information as a substitute for the advice of health care providers, whom they distrust. Individuals who reported more frequent health care visits in the last 2 years were also positively associated with following web-based medical advice (*P*<.001). When individuals used the emergency room or urgent care as their primary source of health care, they were more likely to follow the presented advice (*P*=.04). The sex, race, and income of our respondents were not significantly related to whether they followed web-based medical advice, but education and age were significant predictors. Compared with those without higher education, individuals who had attained at least a bachelor’s degree were significantly less likely to follow web-based medical advice (*P*<.001). In general, younger individuals were less likely to follow web-based medical advice (*P*<.001 for young adults and *P*=.03 for middle-aged adults), which is consistent with previous research that found that younger patients were less likely to believe that providers listened to them [57] and less likely to seek medical care [58].

### Interaction Between Medical Skepticism and Website Trait Influence

In model 2 (Table 2), we additionally examine moderation effects that capture the complex relationship between experimental conditions and medical skepticism. Model 1 demonstrates that individuals reporting higher levels of skepticism were more likely to follow web-based medical advice, regardless of the experimental condition. However, the significant coefficients for our 3 interaction terms indicate that the association between experimental condition and following web-based medical advice does vary by the degree of medical skepticism.

### Physician Authorship × Medical Skepticism

**Figure 3:**
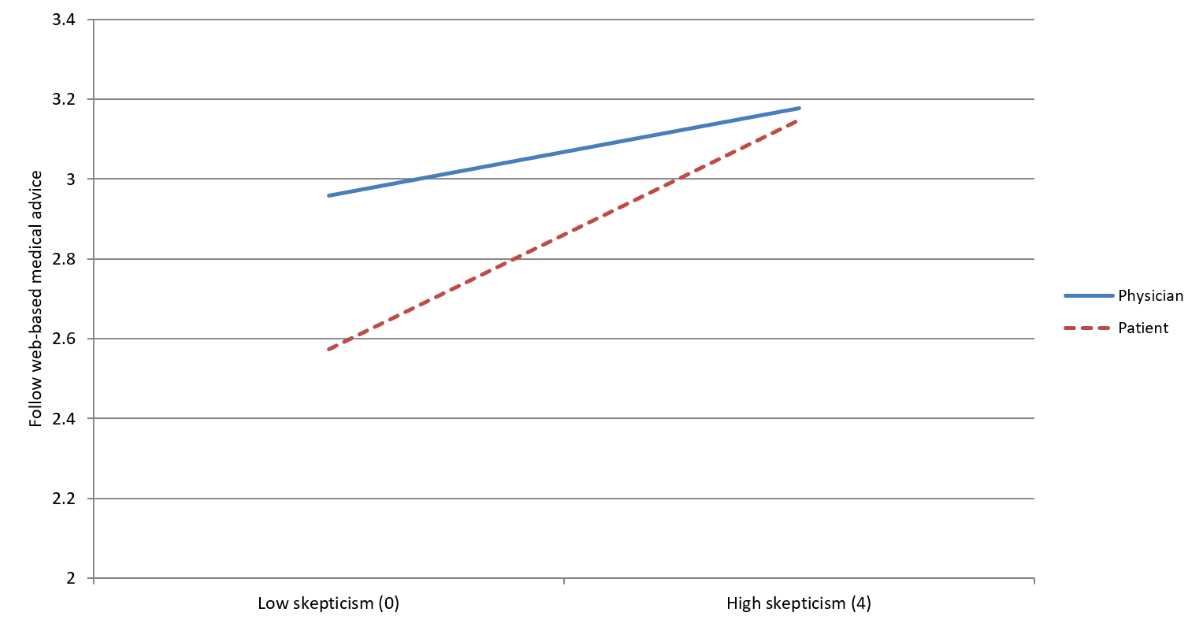
Interaction between medical skepticism and authorship.

Model 1 shows that physician advice (as opposed to patient advice) was more likely to be followed. However, as model 2 and Figure 3 show, this effect was moderated by medical skepticism such that the physician advantage was much smaller among respondents with higher skepticism compared with those with lower skepticism (*P*=.04). Follow-up regression models indicated that the physician authorship advantage was nonsignificant among respondents in the highest tertile of skepticism, but it was relatively large and statistically significant among those in the lowest tertile of skepticism (b=0.202; *P*=.05). Despite this erosion of physician authorship advantage among more skeptical users, individuals across all skepticism levels favored physician-authored web-based material—in no case were respondents more likely to follow web-based medical advice in the experiment after reading a webpage with a patient author.

### Webpage Format × Medical Skepticism

**Figure 4:**
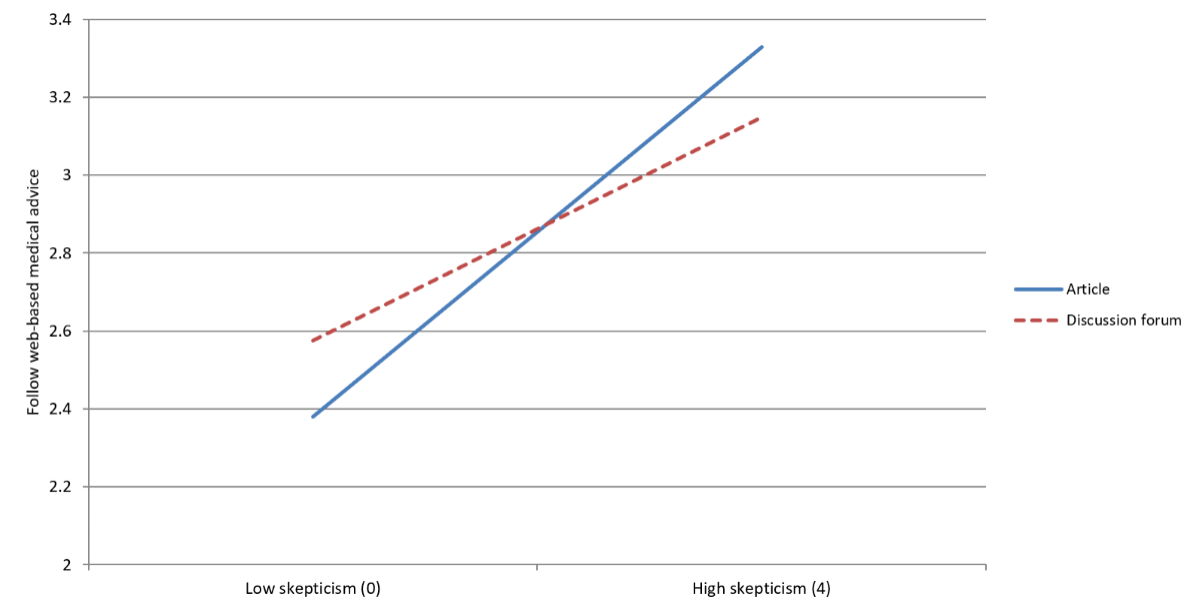
Interaction between medical skepticism and format.

In model 1, the format of the webpage (article vs discussion forum) was not significantly related to whether individuals followed web-based medical advice. However, model 2 and Figure 4 demonstrate that the effect of a webpage format was moderated by medical skepticism (*P*=.03). Those with lower levels of skepticism were more likely to follow web-based medical advice when presented with a discussion forum format. Follow-up regression models indicated a statistically significant discussion forum (vs article) advantage for respondents in the lowest tertile of medical skepticism (b=–0.270; *P*=.008), whereas those in the highest tertile of skepticism significantly preferred an article format (b=0.270; *P*=.008). This suggests that individuals with high levels of medical skepticism are swayed by an article format, but those with low levels of medical skepticism show a preference for a discussion forum format.

### Content Tone × Medical Skepticism

**Figure 5:**
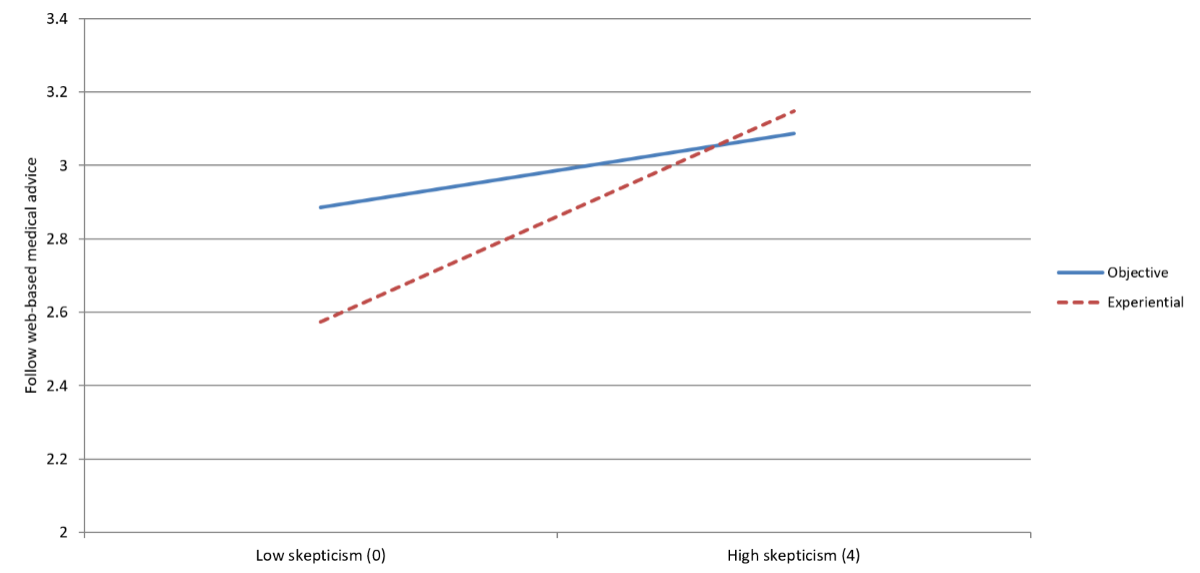
Interaction between medical skepticism and writing style.

Our final interaction term illustrates how individuals with the highest level of medical skepticism have diminished content tone preferences. Model 2 and Figure 5 show how the effect of the content tone was moderated by medical skepticism (*P*=.03). Follow-up regression models demonstrate that the objective tone advantage was statistically significant among respondents in the lowest tertile of skepticism (b=0.240; *P*=.02). On average, people are more likely to follow objectively written advice, but that advantage disappears among those with higher skepticism.

## Discussion

### Principal Findings

Our results demonstrate that, within our experimental condition of leg cramp advice, website presentation traits of medical information on the web do matter. Generally, patients are more receptive to medical information authored by a physician and presented in an objective tone, with this advantage diminishing among those with higher skepticism. The website format trait (article format vs discussion forum format) is more complex. Although our findings in model 1 suggest this trait is not a significant influencer when considering all participants on average, Figure 4 helps explain that this is due to different preferences depending on the level of medical skepticism, with low-skepticism participants preferring the discussion forum format and high-skepticism participants preferring the article format. This distinction implies that different website formats may be better suited for audiences of different levels of medical skepticism.

Medical skepticism has been previously identified as an important trait to consider when trying to understand patient behavior [5,6,8,9,59]. Learning about medical conditions and taking a more active role in health decisions may manifest in higher medical self-efficacy and simultaneously make patients more skeptical about physician advice [59]. For example, high levels of medical skepticism were associated with the use of complementary and alternative medical treatments in patients with arthritis [5]. We extend our understanding of medical skepticism and show evidence that it is also important in how people consume web-based medical information. We find that people consume and judge web-based medical advice credibility differently, depending on their level of medical skepticism. Patients with a greater degree of medical skepticism are more likely to follow web-based medical advice, regardless of the website traits. This insight makes sense because they are likely more familiar with getting medical advice or ideas from web-based sources because they conduct their own research instead of seeking out and listening to medical professionals in person. This underscores the ever-deepening power of web-based medical advice on patient behaviors, often at the expense of seeking in-person medical diagnoses.

Furthermore, the preferences of physician authorship over patient authorship and an objective writing tone over an experiential writing tone diminish as medical skepticism increases. This implies that medical skeptics may be more open to a wider variety of web-based medical advice. They are receptive to patient and physician accounts as well as experiential recounts, in addition to objective information. However, patients with a greater degree of medical skepticism find the article format more credible than the discussion forum format, which is opposite of the preferences of those with lower degrees of medical skepticism. This is an interesting finding, and may be due to a sense of legitimacy conveyed in an article format to a population more interested in researching and making their own medical decisions.

This research suggests that medical organizations should consider website traits when designing solutions to communicate medical advice. Physician-authored information written in objective tones in a discussion forum format seems to be a potentially effective combination for individuals with low medical skepticism. We do not formally survey web-based information sources to determine the relative frequency of presentation formats, but this combination seems to be rare, presenting a potentially significant opportunity. Our findings may also be useful to health care professionals because they interact with patients who are likely bringing their own ideas and information gathered from web sources into consultations. Those with higher levels of medical skepticism tend to be less discriminating about website traits, although they find web-based articles more persuasive.

### Limitations and Future Research Directions

The hypothetical situation of a leg cramp scenario might have influenced participants in certain ways that limit the broader generalizability of our insights. The strength of an experiment is the ability to control factors in the participant’s situation, but the experimental scenario may not accurately predict how real-world scenarios unfold [60]. Care was taken to select a medical scenario that (1) was plausible to research independently (ie, not so severe as to need immediate medical attention) and (2) could involve medical treatment advice that was not trivial but did not necessitate physician oversight. We also screened out participants who had previous experience with leg cramps in an attempt to recruit unbiased participants without existing opinions about leg cramp treatments.

Patients read internet content on a variety of devices, including desktop computers, laptops, tablets, and smartphones [61]. Our data reflect this variety, with 54.49% (1259/2305) of the participants accessing the study from mobile devices. Therefore, we believe that our findings are generalizable to a variety of consumer devices used by patients. However, future work would benefit from studying user device preferences more directly to see whether they alter patient acceptance of web-based medical advice.

Our website format distinction was between an article format (static website that the owner controls) and a discussion forum format (dynamic cocreation among multiple users). It is worth noting that there is a plethora of discussion forum styles and blogs, with their own different sets of features and uses. Our design incorporated a simple discussion forum layout, but future research could extend this research question to determine which set of traits and functions in a discussion forum layout distinctly affect patients’ willingness to follow web-based medical advice.

Our study focused on nonchronic ailments for which patients may seek web-based medical advice. Patients who have chronic ailments may exhibit different behaviors regarding their assessment and adherence to web-based medical advice. For example, previous work has examined the importance of web-based communities for patients with chronic diseases [62-64]. Another extension of this research would be to consider a broader decision-making process of the patient. Our experimental design focused only on the participant’s initial assessment of a single isolated webpage. In reality, patients may look at multiple webpages to form opinions.

### Conclusions

Although already prevalent, the number of patients conducting their own web-based medical research increases every year [13,15]; yet, not enough is known about what makes a medical website persuasive to the average patient. Our research identifies three website traits (authorship, format, and tone) relevant to patient credibility assessments and provides evidence about how these traits influence patients. Furthermore, we extend the conversation about medical skepticism and show how patients with high levels of medical skepticism may interpret website traits to assess medical advice credibility differently.

## Abbreviations

OLS: ordinary least squares
